# The development of a theory and evidence-based intervention to aid implementation of exercise into the prostate cancer care pathway with a focus on healthcare professional behaviour, the STAMINA trial

**DOI:** 10.1186/s12913-021-06266-x

**Published:** 2021-03-25

**Authors:** Rebecca R. Turner, Madelynne A. Arden, Sophie Reale, Eileen Sutton, Stephanie J. C. Taylor, Liam Bourke, Diana M. Greenfield, Dylan Morrissey, Janet Brown, Patrick Doherty, Derek J. Rosario, Liz Steed

**Affiliations:** 1 Allied Health Professionals, Radiotherapy and Oncology, Sheffield Hallam University, Sheffield, UK; 2 Centre for Behavioural Science and Applied Psychology (CeBSAP), Sheffield Hallam University, Sheffield, UK; 3 Population Health Sciences, University of Bristol, Bristol, UK; 4 Institute for Population Health Sciences, Queen Mary, University of London, London, UK; 5 Specialised Cancer Services, Sheffield Teaching Hospital NHS Foundation Trust, Sheffield, UK; 6 Department of Oncology and Metabolism, University of Sheffield, Sheffield, UK; 7 Sports and Exercise Medicine, William Harvey Research Institute, School of Medicine and Dentistry, Queen Mary, University of London, London, UK; 8 Physiotherapy Department, Barts Health NHS Trust, London, UK; 9 Department of Health Sciences, University of York, York, UK; 10 Department of Urology, Sheffield Teaching Hospitals, Sheffield, UK

**Keywords:** Prostate cancer, Androgen deprivation therapy, Intervention development, Exercise, Healthcare professionals, Stakeholders, Behaviour change wheel, Medical Research Council, Theoretical domains framework, Patient and public involvement

## Abstract

**Background:**

Twice-weekly supervised aerobic and resistance exercise for 12 weeks reduces fatigue and improves quality of life in men on Androgen Deprivation Therapy for prostate cancer. Despite the National Institute for Health and Care Excellence (NICE) proposing this as standard of care, it does not routinely take place in practice. Healthcare professionals are in a prime position to deliver and integrate these recommendations. A change in the behaviour of clinical teams is therefore required.

In this paper, we describe the development of a training package for healthcare professionals using theory and evidence to promote delivery of such recommendations as standard care.

**Methods:**

The intervention development process was guided by the Medical Research Council guidance for complex interventions and the Behaviour Change Wheel. Target behaviours were identified from the literature and thirty-five prostate cancer care healthcare professionals (including oncologists, consultant urologists, clinical nurse specialists, physiotherapists, general practitioners and commissioners) were interviewed to understand influences on these behaviours. The Theoretical Domains Framework was used to identify theoretical constructs for change. Behaviour change techniques were selected based on theory and evidence and were translated into intervention content. The intervention was refined with the input of stakeholders including healthcare professionals, patients, and exercise professionals in the form of rehearsal deliveries, focus groups and a workshop.

**Results:**

Seven modifiable healthcare professional target behaviours were identified to support the delivery of the NICE recommendations including identifying eligible patients suitable for exercise, recommending exercise, providing information, exercise referral, providing support and interpret and feedback on progress. Ten domains from the Theoretical Domain’s Framework were identified as necessary for change, including improving knowledge and skills, addressing beliefs about consequences, and targeting social influences. These were targeted through twenty-two behaviour change techniques delivered in a half-day, interactive training package. Based on initial feedback from stakeholders, the intervention was refined in preparation for evaluation.

**Conclusions:**

We designed an intervention based on theory, evidence, and stakeholder feedback to promote and support the delivery of NICE recommendations. Future work will aim to test this training package in a multi-centre randomised trial. If proven effective, the development and training package will provide a template for replication in other clinical populations, where exercise has proven efficacy but is insufficiently implemented.

**Supplementary Information:**

The online version contains supplementary material available at 10.1186/s12913-021-06266-x.

## Background

Prostate cancer is common, with over 1,276,000 men diagnosed worldwide in 2018 [1]. Approximately half of all men diagnosed with prostate cancer will undergo androgen deprivation therapy (ADT), otherwise referred to as medical castration [2]. ADT is administered either in isolation or in conjunction with another treatment such as radiotherapy [3] or chemotherapy [4]. Whilst ADT is effective in treating prostate cancer, rapid withdrawals of androgens are associated with debilitating side-effects including: fatigue [5], weight gain [6], loss of muscle mass [7], loss of bone mineral density [8], sexual dysfunction [9], cardiovascular morbidity and mortality [10] and psychological distress [11], all resulting in a reduced quality of life (QoL) [12].

Evidence suggests that non-pharmacological interventions, predominantly involving aerobic and resistance exercise, are the only safe and beneficial treatments to improve several of the side-effects of ADT; in particular fatigue, muscle loss, cardiovascular disease risk, and result in improved QoL [13–16]. Guidelines exist internationally recommending such interventions as an essential part of prostate cancer care [17–19]. For instance, in 2014, the UK National Institute for Health and Care Excellence (NICE) incorporated a recommendation in prostate cancer management guidelines stating all men starting ADT should be offered 12 weeks of twice-weekly supervised aerobic and resistance exercise to reduce fatigue and improve QoL (NG131 1.4.19) [20]. Despite this, it has been shown that these recommendations are rarely implemented in usual care, with only 2% of NHS trusts self-reporting delivery of these recommendations in 2018 [21].

Healthcare professionals’ (HCPs) behaviours are central to ensuring that recommendations are supported and delivered. Specifically, a key worker role (commonly a clinical nurse specialist) coordinates patient care and provides further support where applicable. The key worker is well placed to intervene and offer exercise recommendations and referral. This is also in line with their current role, as there is growing recognition of the need to provide holistic care and to promote self-management strategies such as exercise. This is reflected in the NHS long-term plan [22], Macmillan Competency Framework for nurses (MCFN) [23] and the cancer recovery package [24]. Additionally, men with prostate cancer on ADT report a willingness to exercise [25] but a desire for their HCP to endorse exercise and make exercise referrals to enable access to services [21].

Specifically clinical nurse specialists report several barriers to discussing exercise with cancer survivors, including a lack of awareness of clinical exercise recommendations [26], a lack of adequate support structures and potential risks to patients [27]. Furthermore, HCPs identify a lack of time during consultations to discuss exercise [28] and a lack of confidence in providing behavioural support to cancer survivors about exercise [29]. Additionally, several negative assumptions held by HCPs concerning exercise safety, patient capabilities and patient motivation to exercise have been reported [30].

Some large-scale approaches exist to support HCPs to provide exercise recommendation and overcome perceived barriers. For example Public Health England (PHE) provides free training to HCPs across England to encourage HCPs to promote physical activity [31]. Further an initiative, Make Every Contact Count (MECC) [32], provides HCPs with online resources to improve clinical communications skills around exercise. However these approaches have varied in implementation success [33] and are yet to be fully evaluated. In addition, they lack a whole multi-disciplinary clinical team approach to training and do not provide links with an exercise referral scheme, something HCPs report as a barrier to broaching exercise with patients, as they want to be able to offer something tangible to patients [28].

At present there are no formally recognised whole team training programmes for HCPs to support the integration of the NICE NG131 1.4.19 recommendations into the prostate cancer care pathway. We intend to facilitate implementation of these NICE recommendations into practice, starting with HCP behaviour change, as part of an ongoing research programme STAMINA (Supported exercise TrAining for Men wIth prostate caNcer on Androgen deprivation therapy). STAMINA aims to develop, integrate, and evaluate a pathway working in partnership between the NHS and Nuffield Health (NH) to provide exercise in the community to men with prostate cancer on ADT as part of their treatment. Training for the exercise professionals responsible for delivering the exercise prescription at NH has been developed, following similar principles to those for the HCPs.

This paper describes the methods and outcomes from the development and refinement of a theory and evidence-based training package (as part of the STAMINA programme) to facilitate HCPs to provide exercise recommendations, support and referrals in line with NICE recommendations. This work was guided by formalised intervention development approaches; the Medical Research Council (MRC) guidance for developing and evaluating complex interventions [34] and the Behaviour Change Wheel (BCW) [35]; drawing upon the application of theory using the Theoretical Domains Framework (TDF) [36], and stakeholder input [37] guided by Normalisation Process Theory (NPT) [38]. The MRC guidance for developing and evaluating complex interventions emphasises the importance of integrating theory and the best available evidence to develop complex interventions. The BCW builds upon the MRC guidance and offers a practical guide of how to develop theory and evidence based intervention [35]. The BCW is a systematic tool for designing complex interventions for researchers to understand behaviour/s, identify the theoretical process to facilitate behaviour change and specify intervention content. At the core of the BCW is an understanding that ‘Behaviour’ is influenced by an individual, or systems, ‘Capability, Opportunity and Motivation’ (COM-B model). The COM-B elements can further be mapped to theoretical constructs using the Theoretical Domains Framework (TDF) [36]. The TDF is a comprehensive framework of 14 theoretical domains synthesised from 128 theoretical constructs and 33 behavioural or behaviour change theories [39] and was developed to support the implementation of new healthcare practices requiring behaviour change. Whilst the COM-B model [35] and TDF [36] offer insight into behaviour and behaviour change, NPT specifically aims to understand why key mechanisms promote or inhibit the implementation and integration of interventions into healthcare.

The aim of this research was to develop a theory and evidence-based training package, with stakeholder input for HCPs working in secondary prostate cancer care, to support the delivery of the NICE recommendations NG131 1.4.19. The broader aim of this paper is to provide a transparent overview of how to systematically develop a HCP intervention drawing on theory, evidence, and stakeholder involvement.

## Methods

**Fig. 1:**
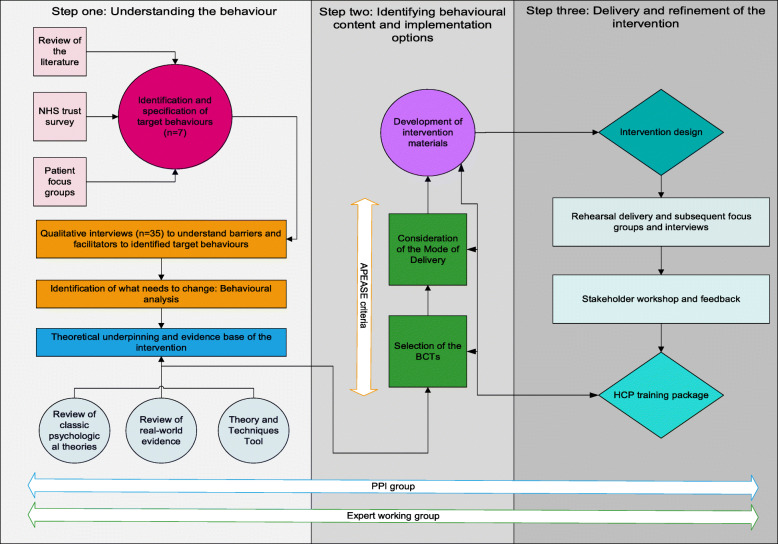
Overview of the intervention development process

The intervention was developed in accordance with the MRC guidance for the development of complex interventions [18, 22] and the BCW [20]. The intervention was refined through an iterative and dynamic process based on evidence, theory and feedback from intervention recipients, patient and public involvement (PPI) group members, stakeholders and a multi-disciplinary expert working group with expertise in behaviour change, complex intervention development, prostate cancer and exercise trials, healthcare, and qualitative methodologies (see Fig. 1).

UK NHS (15/SW/0260) and University ethical boards provided approval for this study approved for Step one. Further ethical approval was granted 23rd October 2018 by North West - Liverpool East Research Ethics Committee. REC reference: 18/NW/0738 / IRAS project ID: 254343 for Step three.

### Step one: understanding the behaviour

#### Identification and selection of target behaviours

A list of behaviours conducted by HCPs that support the delivery of the NICE guideline (NG131 1.4.19) were generated by reviewing i) a Cochrane review describing intervention characteristics associated with successful uptake and improvement of exercise behaviour in cancer survivors [40], ii) literature around the HCP role in discussing exercise in cancer survivors, (see Background), iii) qualitative findings of the experiences of men with prostate cancer on ADT and their beliefs about exercise [21].

Potential behaviours were then presented to the expert working group and PPI group. A consensus decision was taken on which behaviours should be the target of the intervention based on the following criteria as guided by the BCW [35]: i) robust evidence of importance, ii) likely to be able to applied in an existing clinical pathway, iii) amenability to change.

#### Specification of behaviours

To understand the role of HCPs in target behaviours, the provision of exercise and current standard care within the prostate cancer pathway a survey was sent to all NHS secondary care trusts. For each target behaviour it could then be specified (through consensus with the expert working group and PPI group) *who* needs to deliver the behaviour, *what* does a person need to do differently, *when* will it happen and *where* will it take place. *How often* this behaviour would be performed and *with whom* were also considered.

#### Identifying what needs to change

Semi-structured interviews with HCPs (including oncologists, consultant urologists, clinical nurse specialists, physiotherapists, general practitioners and commissioners) working within prostate cancer care were conducted, (see Bourke et al. (2018)) [21]. HCPs were purposively recruited to represent those working within varied roles in the NHS advanced prostate cancer care pathway. The topic guide was based on the TDF (see Additional file 1) and interviews were analysed using a deductive framework [41] guided by the TDF [42] in NVivo V9. In addition, an inductive analytical approach captured any data on behavioural determinants outside the TDF framework. The lead researcher (RT) examined each transcript, coding according to the framework and referring to the coding manual. Text could be attributed to more than one domain, where applicable. Subsequently two additional researchers (ES, LS) independently coded a sub-set of the five same transcripts for comparison, discrepancies were discussed and resolved by consensus with reference to the coding manual. Data was then summarised into core themes under the TDF domains.

Once key themes were identified under the TDF domains, a behavioural analysis for each target behaviour was carried out, this involved identifying what theoretical constructs (e.g. beliefs about consequences) from the TDF needed to change for each specific behaviour to occur (e.g. recommendation of exercise).

#### Theoretical underpinning of the intervention

Psychological theories of behaviour change were reviewed, that incorporated the constructs of interest within the TDF. This provided insight in to how best to apply these theories to the current context and advance our understanding of the likely mechanisms of change. Additionally, the literature around successful ways to encourage professional behaviour change were also reviewed. In line with MRC guidance, a logic model was developed to present the theoretical underpinning of the intervention [43, 44]. This was refined throughout the intervention development process with stakeholder input.

### Step two: identifying behavioural content and implementation options

#### Behaviour change techniques

Having hypothesised the theoretical underpinning to achieve change in the target behaviour, content for the intervention was developed and was guided by the selection of behaviour change techniques (BCTs). BCTs are defined as “*an active component of an intervention designed to change behaviour”* [45]. We used the labels and detailed definitions of BCTs in accordance with the Behaviour Change Technique Taxonomy version 1 (BCTTv1) [45]. BCTs were chosen from i) recent systematic reviews that have highlighted specific BCTs that have been shown to be effective when promoting professional behaviour change specifically in HCPs, ii) selecting BCTs that are proposed by the theories the intervention is underpinned with, iii) using the theory and techniques online tool [46]. Similarly, BCTs to be delivered by the HCPs to support exercise behaviour in men with prostate cancer on ADT were identified from evidence [40, 47] and theory. The APEASE criteria^1^ (Acceptability, Practicability, Effectiveness, Affordability, Side-effects, and Equity) was considered throughout this process when making decisions regarding intervention development.

#### Mode of delivery

Once the BCTs had been identified, behavioural content was then developed. The mode of delivery of the overall intervention in the form of a training package was in accordance with preferences expressed by HCPs in semi-structured interviews (see Step one). Intervention materials were created, alongside training manuals for the HCPs and facilitators manuals for the training providers.

### Step three: delivery and refinement of the intervention

Refinement of the intervention included multiple, iterative, steps as presented in Fig. 1.

#### Rehearsal delivery and subsequent focus groups and interviews

The training intervention was delivered to one NHS prostate cancer clinical team. HCPs attended the half-day rehearsal delivery of training sessions, which were carried out face-to-face and delivered by researchers. Focus groups and interviews were conducted immediately after the rehearsal deliveries to gain feedback from HCPs on the intervention as initially designed, intervention materials and delivery. The topic guide was based on Kirkpatrick (1997) [48] (see Additional file 1). Kirkpatrick (1977) argues there are four processes to evaluate in training programmes. These are reaction; learning; behaviour and results. The topic guide was based on the concept’s reaction and learning. Reaction is concerned with understanding how participants feel about the training programme. Learning is to what extent the attendees have learnt something new such as skills. The focus groups were facilitated by a team member independent of the training team to reduce the risk of bias, audio-recorded and transcribed. Inductive thematic analysis [49] was carried out to analyse the focus groups transcripts using NVivo 9. One independent researcher coded the transcripts, with a subset coded by another researcher, coding was compared face-to-face and any disagreements were resolved by a discussion with a senior qualitative researcher. Key themes and sub themes were identified to inform the refinement of the intervention.

#### Stakeholder workshop and feedback

Following the rehearsal delivery, see Fig. 1, the intervention and intervention materials were presented at a one-day stakeholder workshop in line with Compass Guidance [50]. Participants were identified via contacts who had expressed an interest in the programme via the previous National Institute of Health Research programme development grant, existing clinical and professional networks, research participant representative networks, national charity representatives and our PPI group. The stakeholder workshop ran in the format of presentations from the research team, presenting ‘key uncertainties’ to the group. Small group discussions (by table) were facilitated by a member of the research team, with feedback to the whole group. Discussion was guided by a broad topic guide based on the Normlisation Process Theory [38], (see Additional file 1). Feedback from the stakeholder workshop was collated and analysed using inductive thematic analysis [49]. Field notes from observations, informal discussions and the working groups were also taken throughout the stakeholder workshop by the researchers. Field notes were individually recorded collated, combined, and circulated to attendees as a form of respondent validation.

## Results

### Step one: understanding the behaviour

#### Identification, selection and specification of target behaviours

Seven HCP target behaviours were identified for change and specified behaviourally, as reported in Table 1.

**Table 1:** Specification of the HCP target behaviours selected to focus upon in the intervention

Target behaviour	Who?	Where and When? Secondary care
1. Identify patient as suitable for exercise	Consultant and/or key worker	At any point within the pathway
2. Recommend exercise training	Consultant and/or key worker	At any point within the pathway
3. Provide patient with information pack and materials	Keyworker	At the point of an exercise referral
4. Discuss barriers and facilitators around exercise training, provide support using BCTs	Keyworker	At the point of an exercise referral
5. Make referral for exercising training	Keyworker	At any point within the pathway
6. Read and interpret exercise progress report	Keyworker	Follow-up appointment
7. Provide feedback to the patient on the exercise progress report	Keyworker	Follow-up appointment

#### Identification of who may perform behaviour

Seventy-two HCPs from secondary care completed the NHS trust survey. Four significant touchpoints of care were identified; 1) diagnosis, 2) initiation of treatment, 3) treatment delivery and 4) follow up. These points of care represent opportunities for the identified HCPs to intervene.

#### Identifying what needs to change

Thirty-five HCPs representing different disciplines (Consultant Urologists, Medical Oncologists, Clinical Oncologists, Clinical Nurse Specialist, General practitioners, Clinical commissioners, and Physiotherapists) within the NHS prostate cancer care pathway took part in the interviews. Ten out of fourteen TDF domains were identified during the analysis as influencing the seven identified behaviours (see Additional file 2) all in line with NG131 1.4.19 recommendations. These ten domains were: *knowledge, behavioural regulation, memory, attention and decision processes, skills, beliefs about capabilities, beliefs about consequences, social/professional role and identity, emotion, environmental context and resources* and *social influences*. Each was a target of the intervention. Full details of the main findings are presented in Bourke et al., (2018) [21].

#### Theoretical underpinning of the intervention

**Fig. 2:**
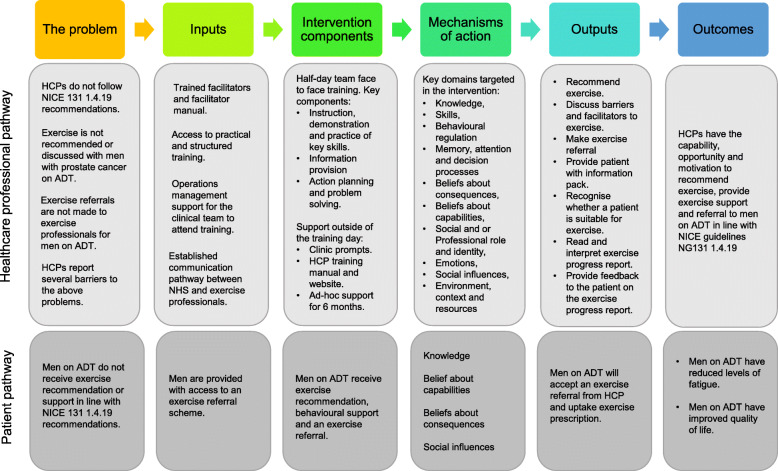
Logic model of the healthcare professional and patient intervention

The intervention development team identified key theoretical domains from the TDF to target in the intervention. To expand understanding of these psychological theories were further drawn upon. These were Social Cognitive Theory [51], the Necessity and Concerns framework [52] and Theories of Habit [53]. Social Cognitive Theory [51] highlights the importance of using well-established techniques such as mastery, vicarious experiences and modelling to develop skills and improve self-efficacy. SCT [51] also highlights the importance of others such as peers when changing behaviours. Ensuring HCPs had support from colleagues and management was critical and training as a clinical team was seen as important. The Necessity and Concerns framework [52] demonstrates the importance of providing information and addressing worries about a treatment (e.g. exercise) to make adherence more likely. Habit has an important role in health professional behaviour and theories of habit [53] were drawn upon to inform strategies such as clinic prompts and action planning. A logic model was developed based on the underlying theory as shown in Fig. 2.

### Step two: identify behavioural content and implementation options

#### Behaviour change techniques

Twenty-two BCTs linked to the theoretical domains and psychological theories were identified as shown in Table 2.

**Table 2:** Overview of the HCP training package including behaviour change techniques used and mode of delivery

Modules	Mode of delivery	BCTs (coded in line with BCTTV1)	Associated TDF domain
1. Overview of the project	• Use of an importance ruler to assess HCP perceptions on exercise. • Information presented about the project, NICE recommendations, patient experiences with exercise and proposed new HCP roles. • Information presented as patient vignettes, videos, written text, prompts for clinic use and links to further reading. • Problem solving task in relation to new HCP roles such as providing exercise referrals.	Problem solving (1.2)	Beliefs about capabilities
		Social support (Unspecified) (3.1)	Skills, Behavioural regulation, and Social influences
		Information about health consequences (5.1)	Knowledge and Beliefs about consequences
		Information about social and environmental consequences (5.3)	Knowledge
		Information about emotional consequences (5.6)	Knowledge and Beliefs about consequences
		Prompts/cues (7.1)	Memory and Environmental resources and context
		Credible source (9.1)	Skills, Social/Professional role and identity and Social Influences
		Adding objects to the environment (12.5)	Memory, attention and decision processes and Environment resources and context
2. Prostate cancer and exercise – the evidence	• Information presented about the evidence base for exercise in prostate cancer. • Information presented via videos, written text, patient case studies, handouts of scientific papers and links to further reading. • Case study task in relation to reducing assumptions about patient’s suitability for exercise.	Social support (Unspecified) (3.1)	Skills, Behavioural regulation, and Social influences
		Information about health consequences (5.1)	Knowledge and Beliefs about consequences
		Information about social and environmental consequences (5.3)	Knowledge
		Information about emotional consequences (5.6)	Knowledge and Beliefs about consequences
		Information about others’ approval (6.3)	Social/Professional role and identity and Social influences
		Credible source (9.1)	Skills, Social/Professional role and identity and Social Influences
		Reduce negative emotions (11.2)	Beliefs about capabilities and emotions
3. Discussing exercise as a healthcare professional	• Discussion around pros and cons of discussing lifestyle factors with this patient group and problem-solving task. • Information on the teachable moment, patient views of HCPs discussing lifestyle, on new roles for HCPs and procedures. • Demonstrations of discussions of exercise with patients. • Information presented as patient vignettes, videos, written text, prompts for clinic use and links to further reading. • Action planning task as a team.	Problem solving (1.2)	Beliefs about capabilities
		Action planning (1.4)	Behaviour regulation
		Social support (unspecified) (3.1)	Skills, Behavioural regulation, and Social influences
		Instruction on how to perform the behaviour (4.1)	Knowledge, Skills and Beliefs about capabilities
		Information about health consequences (5.1)	Knowledge and Beliefs about consequences
		Information about social and environmental consequences (5.3)	Knowledge
		Information about emotional consequences (5.6)	Knowledge and Beliefs about consequences
		Demonstration of behaviour (6.1)	Skills and Beliefs about capabilities
		Information about others’ approval (6.3)	Social/Professional role and identity and Social Influences
		Prompts/cues (7.1)	Memory, attention and decision processes and Environment resources and context
		Habit formation (8.3)	Environmental context and resources
		Credible source (9.1)	Skills, Social/Professional role and identity and Social Influences
		Pros and cons (9.2)	Beliefs about consequences
		Comparative imagining of future outcomes (9.3)	Beliefs about consequences
		Adding objects to the environment (12.5)	Memory, attention and decision processes and Environment resources and context
		Verbal persuasion about capability (15.1)	Beliefs about capabilities
		Focus on past success (15.3)	Beliefs about capabilities
4. Skills for supporting people with exercise	• Information about behaviour change and behaviour change theory, introduction to techniques to support behaviour change in this patient group and demonstration of these. • Overview of all the specific BCTs, providing instruction and demonstrations how to deliver them. • Opportunity for role play and feedback tasks in relation to techniques. • Reflections on previous experiences. • Information presented as patient vignettes, diagrams demonstrations, written text, prompts for clinic use and links to further reading.	Feedback on behaviour (2.2)	Skills
		Social support (unspecified) (3.1)	Skills, Behavioural regulation, and Social influences
		Instruction on how to perform the behaviour (4.1)	Skills, Knowledge and Beliefs about capabilities
		Information about health consequences (5.1)	Knowledge and Beliefs about consequences
		Information about social and environmental consequences (5.3)	Knowledge
		Information about emotional consequences (5.6)	Knowledge and Beliefs about consequences
		Demonstration of behaviour (6.1)	Skills and Beliefs about capabilities
		Behavioural practice/rehearsal (8.1)	Skills and Beliefs about capabilities
		Adding objects to the environment (12.5)	Memory, attention and decision processes and Environment resources and context
		Verbal persuasion about capability (15.1)	Beliefs about capabilities
		Focus on past success (15.3)	Beliefs about capabilities
5. The role of exercise professionals	• Discussion of opinions and experience of exercise referral schemes. • Information about NH, services they provide and their exercise professionals. • Information about the exercise prescription for patients and their views. • Information presented via diagrams, videos and written text.	Social support (unspecified) (3.1)	Skills, Behavioural regulation, and Social influences
		Information about health consequences (5.1)	Knowledge and Beliefs about consequences
		Information about social and environmental consequences (5.3)	Knowledge
		Information about emotional consequences (5.6)	Knowledge and Beliefs about consequences
		Information about other approval (6.3)	Social/Professional role and identity and Social Influences
		Credible source (9.1)	Skills, Social/Professional role and identity and Social Influences
6. The exercise referral pathway and communication pathway	• Overview of the processes for referrals and communication. • Information provided via demonstrations, written text and prompts for clinic use. • Discussion around providing support at follow-up with information provided by written text and patient vignettes. • Opportunity for role play and feedback tasks in relation to techniques.	Problem solving (1.2)	Beliefs about capabilities
		Feedback on behaviour (2.2)	Skills
		Social support (unspecified) (3.1)	Skills, Behavioural regulation, and Social influences
		Instruction on how to perform the behaviour (4.1)	Skills, Knowledge and Beliefs about capabilities
		Information about health consequences (5.1)	Knowledge and Beliefs about consequences
		Information about social and environmental consequences (5.3)	Knowledge
		Information about emotional consequences (5.6)	Knowledge and Beliefs about consequences
		Prompts/cues (7.1)	Memory, attention and decision processes and Environment resources and context
		Behavioural practice/rehearsal (8.1)	Skills and Beliefs about capabilities
		Habit formation (8.3)	Environment context and resources
Intervention support	• The use of prompts within clinics, feedback on behaviour such as referrals via email or telephone and the use of a screening log to self-monitor behaviour.	Feedback on behaviour (2.2)	Skills
		Self-monitoring of behaviour (2.3)	Skills and Behaviour regulation
		Feedback on outcome(s) of behaviour (2.7)	Skills
		Prompts/cues (7.1)	Memory, attention and decision processes and Environment resources and context
		Habit formation (8.3)	Environment context and resources
		Adding objects to the environment (12.5)	Memory, attention and decision processes and Environment resources and context

#### Mode of delivery

An interactive, skills-based, face-to-face, half-day training package, including six modules was developed based around these BCTs (see Table 2). Two levels of training (Level one and Level two) were formed. Level one applied to all HCPs and included identifying suitable patients (target behaviour one) and recommending exercise (target behaviour two). Level two provided greater depth on discussing exercise and making referrals (target behaviours three, four, five, six and seven) and was primarily relevant for key workers, although open to all interested parties.

HCPs were additionally taught to deliver eight BCTs to patients in order to provide behavioural support around exercise including Problem solving, Social Support (unspecified), Information about health consequences, Information about social and environmental consequences, Information about emotional consequences, Information about others’ approval, Pros and Cons and Verbal persuasion about capability.

### Step three: delivery and refinement of the intervention

The intervention was refined following feedback from HCPs, stakeholders, and PPI group members. A summary of the main changes is provided below, with further detail in Additional files 3 and 4.

#### Rehearsal delivery and subsequent focus groups and interviews

Eight HCPs (Consultant Urologists, Clinical Nurse Specialists, and staff nurses) took part in the rehearsal deliveries and all participated in subsequent focus groups or interviews. Four themes were identified: *1) Content –* Training would benefit from patient case studies, task-based exercise and clearer messages about roles; *2) Duration –* The balance should be drawn between the training being long enough to engage HCPs but not too burdensome, with half a day being acceptable; *3) Mode of delivery –* face to face training was strongly endorsed in preference to online; *4) Barriers to the intervention –* HCPs need to be supported by management to attend the training. All suggestions were deemed important and strategies to implement these changes into the training package were made such as engaging with operational teams alongside HCPs to promote the importance of HCPs attending training (see Additional file 3).

#### Stakeholder workshop and feedback

Twenty-eight stakeholders took part in the workshop. These included HCPs (*n* = 3) academics (*n* = 3), researchers, PPI members (*n* = 8), members of local charities (*n* = 2), commissioners (*n* = 3), senior NH staff (*n* = 3) and exercise professionals (*n* = 6). Feedback was mapped onto the four domains of the Normalisation Process Theory (see Additional file 4): 1) *Coherence* – it was viewed as important to include the evidence-base of the wider intervention; 2) *Cognitive participation* – Training sessions would benefit from including more discussion-based activities, clear information and the use of patient stories; 3) *Collective action –* Organisation of training should fit with context of HCP day to day work in NHS: flexible timing, flexible delivery to suit role, sufficient notice for training, range of modes of delivery. 4) *Reflexive monitoring* – The inclusion of action planning and problem solving as a clinical team was suggested. The feedback, repeated by several participants, mirrored suggestions from HCPs and was deemed important. In response, strategies were translated into the training package such as including activities to action plan as a clinical team.

The final intervention is presented in Additional file 5 in line with TIDieR recommendations.

## Discussion

This paper describes a systematic, evidence and theory-based approach to the development of an intervention for HCPs to support the delivery of NICE recommendations (NG131 1.4.19). The intervention was refined through an iterative and dynamic process based on evidence, theory and feedback from HCPs, PPI group members, stakeholders and a multi-disciplinary expert working group. The intervention development process and outcomes may provide a template for HCPs to recommend and support exercise for other long-term conditions, where exercise is effective but under-utilised.

We developed an in-depth understanding of patient and professional needs and identified key modifiable behaviours for change, which is essential when developing complex behavioural interventions [35, 37]. We designed a training package for HCPs to overcome barriers to support the delivery of NICE recommendations (NG131 1.4.19). The intervention focuses upon training the whole clinical team and is tailored to ensure the relevant HCPs are trained, depending upon their role. This approach is often lacking within HCP training packages such as ones from PHE [31, 32] but is critical for a service level change. We worked with HCPs and stakeholders to ensure the training was acceptable, could be delivered within an NHS setting and was complementary to the NHS long-term plan [22], Macmillan Competency Framework for nurses (MCFN) [23] and the cancer recovery package [24]. It was important to ensure the training was aligned with these approaches due to the ever-competing demands within healthcare [54, 55].

The intervention development process through its iterations has been transparent which allows other researchers to observe the complexity of developing behavioural interventions. While the BCW and MRC offered structured direction for intervention development, there are significant gaps within this guidance. Firstly, both approaches highlight the importance of theory in developing interventions, but neither offer explicit guidance on how to select and apply appropriate theories especially when selecting BCTs [56]. To tackle this issue, we had to review potentially relevant classic psychological theories. Aspects of theories were selected to underpin this intervention, these were from Social Cognitive Theory [51], the Necessity and Concerns framework [52] and Theories of Habit [53]. Linking theory and intervention content is challenging and may explain the fundamental issue of interventions being developed without theory [57]. Further research is required to map theoretical constructs derived from theories onto effective intervention content [58]. In addition to theory, BCTs were also selected based on real-world evidence, where BCTs had been found to be effective in HCPs behaviour change. Understanding what techniques have been used, with what effects and in what ‘real-world’ contexts is an essential step to developing an intervention. Furthermore, the work by Carey et al., (2018) was important in the selection of behavioural content. This tool (theory and techniques tool) allowed for us to identify the mechanisms of action, and identify BCTs that were likely to be effective to change behaviour. An important consideration whilst using the theory and techniques tool alongside the BCW is that BCTs which are linked to the intervention functions in the guide as ‘most used’, differ greatly in comparison to the evidence-based theory and techniques tool. At this point, a decision was made to not use the BCW guidance to select behavioural content due to the guide not providing sufficient evidence of effectiveness of behavioural content.

### Strengths and limitations

Context is important for implementation and this is reflected in several theories, models, and frameworks [59]. The process of understanding the determinants of identified behaviours within the specified context, whilst lengthy, is critical for developing a potentially effective intervention. Furthermore, using the NPT [38] to understand these barriers is a strength. The NPT allowed us to consider the wider contextual and organisation issues of implementation.

Whilst, the intervention development team was multi-disciplinary, involving HCPs earlier in the intervention development process (e.g. Identification of target behaviours) would have been advantageous [60, 61]. Examples of work, where the BCW intervention development process has involved intervention users such as clinical teams in partnership with academics have been found to be helpful in improving self-efficacy and giving HCPs the tools to help change practice [61].

### Implications for future research and practice

The NICE131 1.4.19 recommendations are an important starting point to increase knowledge and awareness, but alone they have not changed behaviour of the clinical teams, with no exercise provision being offered for this patient group as standard care [21]. Multiple complex behaviours are required from the HCPs to implement such recommendations and support men with prostate cancer on ADT obtain the benefits of regular, supervised exercise. This paper highlights the key target behaviours required to implement the NICE recommendations NG131 1.4.19 and can provide a template to facilitating HCPs to recommend exercise for other long-term conditions, where exercise is effective but under-utilised.

The training package is reported to the point where it can be delivered in a feasibility study with other components of the STAMINA wider intervention including training of exercise professionals, delivery to patients and development of a communication pathway. Testing of the integration of intervention components is a critical part of the whole system development and it is expected further refinements may be made before piloting and full evaluation in a randomised cluster definitive trial. It is unlikely that the theoretical underpinning of the intervention will change given the robust development to date. However, considerations will be made to the mode of delivery of the training programme and subsequently the patient exercise intervention due to the current coronavirus pandemic, as telehealth is likely to play a role in healthcare [62]. Telehealth could offer a platform for exercise interventions to be delivered remotely [63], the feasibility and acceptability of this will be explored further. Additionally, delivering training face-to-face with social distancing restrictions may be problematic. Decisions will be led by further stakeholder input.

## Conclusion

We have developed an HCP training package to support the integration of NICE recommendations into the prostate cancer care pathway. The approach to the development of the training package provides a template for developing complex behavioural interventions to facilitate HCPs to recommend and support exercise for other long-term conditions, where exercise is effective but under-utilised. The training package is to be further refined and trialled in a multi-centre cluster randomised trial in the UK.

## Supplementary Information

**Additional file 1.** Topic guides. This file contains three topic guides that were used for primary qualitative data collection in the intervention development and refinement process 1) Focus groups pre-rehearsal delivery to healthcare professionals, 2) Focus groups post-rehearsal delivery to healthcare professionals and 3) Round table discussion during the stakeholder workshop.

**Additional file 2.** Behavioural diagnosis of the seven target behaviours. This file contains an overview of the main barriers to delivery of the seven target behaviours, mapped onto the Theoretical Domains Framework.

**Additional file 3.** Feedback on the healthcare professional intervention following the rehearsal delivery. This file provides feedback on the intervention (training package) following rehearsal delivery to healthcare professionals. Feedback is collated into key themes and mapped onto the APEASE criteria.

**Additional file 4.** Feedback on the healthcare professional intervention from the stakeholder workshop. This file provides feedback on the intervention (training package) following the presentation to stakeholders and roundtable discussions. Feedback is collated into key themes, mapped onto the Normalisation Process Theory and APEASE criteria.

**Additional file 5.** Template for Intervention Description and Replication (TIDieR) of the healthcare professional intervention. This file provides an overview of the healthcare professional intervention (training package).

## Data Availability

The datasets used and/or analysed during the current study are available from the corresponding author on reasonable request.

## Abbreviations

APEASE: **‘**Affordability’, ‘practicability’, ‘effectiveness’, ‘acceptability’, ‘safety/side-effects’ ‘equity’
BCT: Behaviour change technique
BCW: Behaviour change wheel
COM-B: ‘Capability’, ‘opportunity’, ‘motivation’ = ‘behaviour’
HCP: Healthcare professionals
MRC: Medical research council
NICE: National institute for health and care excellence
NH: Nuffield health
NPT: Normalisation process theory
PPI: Patient and public involvement
STAMINA: Supported exercise training for men with prostate cancer on androgen deprivation therapy
TDF: Theoretical domains framework

## Footnotes

1 Affordability refers to the cost of the intervention and this being within an acceptable budget, Practicability refers to whether an intervention can be delivered as designed to the target populations, Effectiveness and cost-effectiveness refers to the effect size of the intervention in relation to the desired objectives in a real-world context and whether this is cost-effective. Acceptability refers to the extent to which an intervention is found to be appropriate by stakeholders and intervention users. Side-effects/safety includes unintended consequences of the intervention and Equity refers to the extent to which an intervention impacts upon disparities in standard of living, health, and well-being between different sectors of society.

## References

[1] Bray F, Ferlay J, Soerjomataram I, Siegel RL, Torre LA, Jemal A (2018). Global cancer statistics 2018: GLOBOCAN estimates of incidence and mortality worldwide for 36 cancers in 185 countries. CA Cancer J Clin.

[2] Sharifi N, Gulley JL, Dahut WL (2005). Androgen deprivation therapy for prostate Cancer. JAMA.

[3] Mason MD, Parulekar WR, Sydes MR, Brundage M, Kirkbride P, Gospodarowicz M, Cowan R, Kostashuk EC, Anderson J, Swanson G, Parmar MKB, Hayter C, Jovic G, Hiltz A, Hetherington J, Sathya J, Barber JBP, McKenzie M, el-Sharkawi S, Souhami L, Hardman PDJ, Chen BE, Warde P (2015). Final report of the intergroup randomized study of combined androgen-deprivation therapy plus radiotherapy versus androgen-deprivation therapy alone in locally advanced prostate Cancer. J Clin Oncol.

[4] James ND, Sydes MR, Clarke NW, Mason MD, Dearnaley DP, Spears MR (2016). Addition of docetaxel, zoledronic acid, or both to first-line long-term hormone therapy in prostate cancer (STAMPEDE): survival results from an adaptive, multiarm, multistage, platform randomised controlled trial. Lancet (London, England).

[5] Walker LM, Tran S, Robinson JW (2013). Luteinizing hormone–releasing hormone agonists: a quick reference for prevalence rates of potential adverse effects. Clin Genitourinary Cancer.

[6] Braunstein LZ, Chen M-H, Loffredo M, Kantoff PW, D'Amico AV (2014). Obesity and the odds of weight gain following androgen deprivation therapy for prostate Cancer. Prostate Cancer.

[7] Haseen F, Murray LJ, Cardwell CR, O'Sullivan JM, Cantwell MM (2010). The effect of androgen deprivation therapy on body composition in men with prostate cancer: systematic review and meta-analysis. J Cancer Surviv.

[8] Edmunds K, Tuffaha H, Galvão DA, Scuffham P, Newton RU (2020). Incidence of the adverse effects of androgen deprivation therapy for prostate cancer: a systematic literature review. Support Care Cancer.

[9] Ng E, Woo HH, Turner S, Leong E, Jackson M, Spry N (2012). The influence of testosterone suppression and recovery on sexual function in men with prostate cancer: observations from a prospective study in men undergoing intermittent androgen suppression. J Urol.

[10] Bourke L, Chico TJ, Albertsen PC, Hamdy FC, Rosario DJ (2012). Cardiovascular risk in androgen suppression: underappreciated, under-researched and unresolved. Heart.

[11] Donovan KA, Walker LM, Wassersug RJ, Thompson LMA, Robinson JW (2015). Psychological effects of androgen-deprivation therapy on men with prostate cancer and their partners. Cancer.

[12] Cheung AS, de Rooy C, Hoermann R, Lim Joon D, Zajac JD, Grossmann M (2017). Quality of life decrements in men with prostate cancer undergoing androgen deprivation therapy. Clin Endocrinol.

[13] Bourke L, Smith D, Steed L, Hooper R, Carter A, Catto J, Albertsen PC, Tombal B, Payne HA, Rosario DJ (2016). Exercise for men with prostate Cancer: a systematic review and meta-analysis. Eur Urol.

[14] Gardner JR, Livingston PM, Fraser SF (2014). Effects of exercise on treatment-related adverse effects for patients with prostate cancer receiving androgen-deprivation therapy: a systematic review. J Clin Oncol.

[15] Keogh JW, MacLeod RD (2012). Body composition, physical fitness, functional performance, quality of life, and fatigue benefits of exercise for prostate cancer patients: a systematic review. J Pain Symptom Manag.

[16] Gilbert SE, Tew GA, Fairhurst C, Bourke L, Saxton JM, Winter EM, Rosario DJ (2016). Effects of a lifestyle intervention on endothelial function in men on long-term androgen deprivation therapy for prostate cancer. Br J Cancer.

[17] Buffart LM, Galvao DA, Brug J, Chinapaw MJ, Newton RU (2014). Evidence-based physical activity guidelines for cancer survivors: current guidelines, knowledge gaps and future research directions. Cancer Treat Rev.

[18] Campbell A, Stevinson C, Crank H (2012). The BASES expert statement on exercise and cancer survivorship. J Sports Sci.

[19] Schmitz KH, Courneya KS, Matthews C, Demark-Wahnefried W, Galvao DA, Pinto BM (2010). American College of Sports Medicine roundtable on exercise guidelines for cancer survivors. Med Sci Sports Exerc.

[20] NICE. National Institute of Health and Care Excellence 2019 [cited 2019 01/07/2019]. Available from: https://www.nice.org.uk/guidance/ng131/chapter/Recommendations#people-having-hormone-therapy.

[21] Bourke L, Turner R, Greasley R, Sutton E, Steed L, Smith D, Brown J, Kelly B, Hulme C, Greenfield D, Persad R, Farrin A, Hewison J, Rosario DJ, on behalf of the STAMINA investigators (2018). A multi-Centre investigation of delivering national guidelines on exercise training for men with advanced prostate cancer undergoing androgen deprivation therapy in the UK NHS. PLoS One.

[22] England N. NHS long-term plan. 2019.

[23] Macmillan. Macmillan Competency Framework (MCFN). 2020.

[24] NCSI. Living with and beyond cancer: taking action to improve outcomes.; 2013.

[25] Gentili C, McClean S, Hackshaw-McGeagh L, Bahl A, Persad R, Harcourt D (2019). Body image issues and attitudes towards exercise amongst men undergoing androgen deprivation therapy (ADT) following diagnosis of prostate cancer. Psychooncology.

[26] Roberts AL, Potts HWW, Stevens C, Lally P, Smith L, Fisher A. Cancer specialist nurses’ perspectives of physical activity promotion and the potential role of physical activity apps in cancer care. J Cancer Surviv. 2019;13:815–28. 10.1007/s11764-019-00801-w PMC6828618 31475306

[27] Pühringer P, editor Physical activity promotion, beliefs, and barriers among Australasian oncology nurses. Oncology Nursing Forum; 2017: Oncology Nursing Society. 10.1188/17.ONF.235-245 28222085

[28] Cantwell M, Walsh D, Furlong B, Moyna N, McCaffrey N, Boran L, Smyth S, Woods C (2018). Healthcare professionals' knowledge and practice of physical activity promotion in cancer care: challenges and solutions. Eur J Cancer Care.

[29] Chisholm A, Hart J, Lam V, Peters S (2012). Current challenges of behavior change talk for medical professionals and trainees. Patient Educ Couns.

[30] Williams K, Beeken RJ, Fisher A, Wardle J (2015). Health professionals' provision of lifestyle advice in the oncology context in the United Kingdom. Eur J Cancer Care (Engl).

[31] Brannan M, Bernardotto M, Clarke N, Varney J (2019). Moving healthcare professionals – a whole system approach to embed physical activity in clinical practice. BMC Med Educ.

[32] PHE. Public Health England: Public Health England, NHS England and Health Education England 2016 [Available from: https://assets.publishing.service.gov.uk/government/uploads/system/uploads/attachment_data/file/769486/Making_Every_Contact_Count_Consensus_Statement.pdf.

[33] Chisholm A, Ang-Chen P, Peters S, Hart J, Beenstock J (2018). Public health practitioners’ views of the ‘making every contact count’ initiative and standards for its evaluation. J Public Health.

[34] Craig P, Dieppe P, Macintyre S, Michie S, Nazareth I, Petticrew M (2008). Developing and evaluating complex interventions: the new Medical Research Council guidance. BMJ.

[35] Michie S, Atkins L, West R. The behaviour change wheel : a guide to designing interventions 2014.

[36] Michie S, Johnston M, Abraham C, Lawton R, Parker D, Walker A (2005). Making psychological theory useful for implementing evidence based practice: a consensus approach. BMJ Qual Saf.

[37] O'Cathain A, Croot L, Duncan E, Rousseau N, Sworn K, Turner KM, Yardley L, Hoddinott P (2019). Guidance on how to develop complex interventions to improve health and healthcare. BMJ Open.

[38] Murray E, Treweek S, Pope C, MacFarlane A, Ballini L, Dowrick C, Finch T, Kennedy A, Mair F, O'Donnell C, Ong BN, Rapley T, Rogers A, May C (2010). Normalisation process theory: a framework for developing, evaluating and implementing complex interventions. BMC Med.

[39] Cane J, O’Connor D, Michie S (2012). Validation of the theoretical domains framework for use in behaviour change and implementation research. Implement Sci.

[40] Turner RR, Steed L, Quirk H, Greasley RU, Saxton JM, Taylor SJ (2018). Interventions for promoting habitual exercise in people living with and beyond cancer. Cochrane Database Syst Rev.

[41] Gale NK, Heath G, Cameron E, Rashid S, Redwood S (2013). Using the framework method for the analysis of qualitative data in multi-disciplinary health research. BMC Med Res Methodol.

[42] Atkins L, Francis J, Islam R, O'Connor D, Patey A, Ivers N (2017). A guide to using the theoretical domains framework of behaviour change to investigate implementation problems. Implement Sci.

[43] Moore GF, Audrey S, Barker M, Bond L, Bonell C, Hardeman W, et al. Process evaluation of complex interventions: Medical Research Council guidance. BMJ. 2015;350:h1258. 10.1136/bmj.h1258. 10.1136/bmj.h1258 PMC4366184 25791983

[44] WK. KF. Logic model development guide. Michigan: Kellogg Foundation WK; 2004.

[45] Michie S, Richardson M, Johnston M, Abraham C, Francis J, Hardeman W, Eccles MP, Cane J, Wood CE (2013). The behavior change technique taxonomy (v1) of 93 hierarchically clustered techniques: building an international consensus for the reporting of behavior change interventions. Ann Behav Med.

[46] Carey RN, Connell LE, Johnston M, Rothman AJ, de Bruin M, Kelly MP, et al. Behavior change techniques and their mechanisms of action: a synthesis of links described in published intervention literature. Ann Behav Med. 2019;53(8):693–707. 10.1093/abm/kay078 PMC6636886 30304386

[47] NICE. Behaviour change: Inidividual approaches 2014 [Available from: https://www.nice.org.uk/guidance/ph49/chapter/1-recommendations#recommendation-9-deliver-very-brief-brief-extended-brief-and-high-intensity-behaviour-change.

[48] Kirkpatrick DL. Evaluating training programs: evidence vs. proof. Train Dev J. 1977;31:9–12.

[49] Braun V, Clarke V (2006). Using thematic analysis in psychology. Qual Res Psychol.

[50] Compass. How to conduct a stakeholder workshop 2014 [cited 2020 3rd May ]. Available from: https://www.thecompassforsbc.org/how-to-guides/how-conduct-stakeholder-workshop.

[51] Bandura A. Social foundations of thought and action. Englewood Cliffs, NJ 1986;1986.

[52] Horne R, Chapman SC, Parham R, Freemantle N, Forbes A, Cooper V (2013). Understanding patients' adherence-related beliefs about medicines prescribed for long-term conditions: a meta-analytic review of the necessity-concerns framework. PLoS One.

[53] Gardner B, Rebar AL. Habit Formation and Behavior Change. Oxford: Oxford University Press; 2019.

[54] Agbassi C, Messersmith H, McNair S, Brouwers M (2014). Priority-based initiative for updating existing evidence-based clinical practice guidelines: the results of two iterations. J Clin Epidemiol.

[55] Dizon DS, Krilov L, Cohen E, Gangadhar T, Ganz PA, Hensing TA, Hunger S, Krishnamurthi SS, Lassman AB, Markham MJ, Mayer E, Neuss M, Pal SK, Richardson LC, Schilsky R, Schwartz GK, Spriggs DR, Villalona-Calero MA, Villani G, Masters G (2016). Clinical Cancer advances 2016: annual report on Progress against Cancer from the American Society of Clinical Oncology. J Clin Oncol.

[56] De Silva MJ, Breuer E, Lee L, Asher L, Chowdhary N, Lund C (2014). Theory of change: a theory-driven approach to enhance the medical research council's framework for complex interventions. Trials.

[57] Prestwich A, Webb TL, Conner M (2015). Using theory to develop and test interventions to promote changes in health behaviour: evidence, issues, and recommendations. Curr Opin Psychol.

[58] Hagger MS, Weed M (2019). DEBATE: do interventions based on behavioral theory work in the real world?. Int J Behav Nutr Phys Act.

[59] Nilsen P (2015). Making sense of implementation theories, models and frameworks. Implement Sci.

[60] Janols R, Lindgren H (2017). A method for co-designing theory-based behaviour change systems for health promotion. Stud Health Technol Inform.

[61] Bull ER, Hart JK, Swift J, Baxter K, McLauchlan N, Joseph S, Byrne-Davis LMT (2019). An organisational participatory research study of the feasibility of the behaviour change wheel to support clinical teams implementing new models of care. BMC Health Serv Res.

[62] Keesara S, Jonas A, Schulman K (2020). Covid-19 and health Care’s digital revolution. N Engl J Med.

[63] Bland KA, Bigaran A, Campbell KL, Trevaskis M, Zopf EM (2020). Exercising in isolation? The role of Telehealth in exercise oncology during the COVID-19 pandemic and beyond. Phys Ther.

