# Supplementary material for: The development of a theory and evidence-based intervention to aid implementation of exercise into the prostate cancer care pathway with a focus on healthcare professional behaviour, the STAMINA trial
Source: BMC Health Serv Res. 2021 Mar 25;21:273. doi: 10.1186/s12913-021-06266-x (PMC7992804; doi:10.1186/s12913-021-06266-x)
Supplement: Supplementary file 1 — Additional file 1. Topic guides. This file contains three topic guides that were used for primary qualitative data collection in the intervention development and refinement process 1) Focus groups pre-rehearsal delivery to healthcare professionals, 2) Focus groups post-rehearsal delivery to healthcare professionals and 3) Round table discussion during the stakeholder workshop. [file 12913_2021_6266_MOESM1_ESM.docx]

**The development of a theory and evidence-based intervention to aid implementation of exercise into the prostate cancer care pathway with a focus on healthcare professional behaviour, the STAMINA trial**

Rebecca R Turner^1^, Madelynne A Arden^2^_,_ Sophie Reale^1^, Eileen Sutton^3^, Stephanie J C Taylor^4^, Liam Bourke^1^, Diana M Greenfield^5,8^, Dylan Morrissey^6,7^, Janet Brown^8^, Patrick Doherty^9^, Derek J Rosario^1,10^ ,Liz Steed^4^ and on behalf of the STAMINA co-investigators.

^1^ Allied Health Professionals, Radiotherapy and Oncology, Sheffield Hallam University, UK

^2^ Centre for Behavioural Science and Applied Psychology (CeBSAP), Sheffield Hallam University, UK

^3^ Population Health Sciences, University of Bristol, UK

^4^ Institute for Population Health Sciences, Queen Mary, University of London, UK

^5^ Specialised Cancer Services, Sheffield Teaching Hospital NHS Foundation Trust

^6^ Sports and Exercise Medicine, William Harvey Research Institute, School of Medicine and Dentistry, Queen Mary, University of London, London UK

^7^ Physiotherapy Department, Barts Health NHS Trust, London, UK

^8^ Department of Oncology and Metabolism, University of Sheffield, UK

^9^ Department of Health Sciences, University of York, UK

^10^Department of Urology, Sheffield Teaching Hospitals, UK

**Corresponding author:** Liz Steed ([e.a.steed@qmul.ac.uk](mailto:e.a.steed@qmul.ac.uk))

### Additional file 1: Topic guides

**File 1a: Semi-structured interview schedule for healthcare professionals to understand barriers to target behaviours based on the theoretical domain’s framework.**

| Semi-structured interview schedule  **Introduction**  Thank you for your time in taking part in this interview. We are interested in your perspective regarding roles, responsibilities and training needs associated with providing supervised exercise programmes for men with prostate cancer on ADT. By supervised exercise, we mean a structured programme of exercise training delivered and overseen by a professional.  We would like to audio record the interviews, but these will be completely confidential, and all data will be anonymised in transcription and analysis. Can you please confirm you have read, understood, and signed the informed consent form and are happy to proceed? |
| --- |
| **Questions** |
| - Can you tell me a little about your current role in the care pathway for men with prostate cancer on androgen deprivation therapy (ADT)? |
| - How do your patients cope with their cancer and ADT? |
| - What are the common adverse effects with this treatment, and which do you feel men find most bothersome? |
| - How would you say being on ADT for prostate cancer affects men’s quality of life? |
| - Are you aware of any non-pharmacological treatment or any supportive programmes designed to improve quality of life for men on ADT? |
| - What do you know about the role of exercise in treating men with PC? (knowledge) |
| - Are you aware of any guidance? Can you tell me what the NICE recommendations are as you see them? (knowledge) |
| - How do you feel about behaviour change strategies like looking at worries and concerns and setting goals? Do you think they have a role in exercise programmes? (beliefs about consequences) |
| **Current guidance recommends men with prostate cancer on ADT have access to supervised exercise which should include an exercise prescription as well as behavioural support such as goal setting and addressing worries and concerns.** |
| - How do you feel about this? |
| - As part of standard NHS practice? |
| - Should this be separate from NHS care? |
| - What is your organisation already doing with regards to exercise for men with prostate cancer? (memory, attention, decision) |
| - Are you currently involved in setting goals with your patients and do you follow up on whether these are achieved or not? |
| - Are you aware of any exercise programmes for other patient groups? How beneficial do you think exercise/exercise programmes would be for your patients with prostate cancer? |
| - Whose role would you see it as to i) make referrals for exercise programmes ii) delivery of exercise? (social/professional role identity) |
| - Should this take place in primary/secondary care/community/outpatient settings (who specifically?)  **Probe* who should introduce idea to patient, delivering exercise (prescription & behaviour change elements), following up with patients the amount of exercise being done. If you do not see it as your role, can you elaborate as to why and who might be better placed?* |
| - Have you been involved in referral or delivery of exercise programmes to any other patient groups in the past? |
| **Our research team are hoping to evaluate how a 12 week, or 12 month, supervised exercise programme could be delivered in the NHS for men on ADT. This will require professionals in your role to support this process. That might involve making referrals, delivering the exercise programme and providing specialised behaviour change support. How would you feel if one or more of these elements became part of your role? (social/professional role identity, emotion)** |
| - What applicable skills do you think you currently have? Do you think you would be able to new relevant learn skills (what training would that need) (skills) |
| - Given training do you think you would feel confident in doing this? (optimism) |
| - How difficult or easy do you think it would be for you to do? (beliefs about capabilities) |
| - Would it be something you would like to do (all/part/none)? (goals) |
| - Would it be compatible with how you see your role? (social/professional role identity) |
| - How do you think your colleagues/seniors e.g. consultants/managers would respond, would this help or be a problem? (social influences) |
| - Would there be capacity to support a 12 week or 12-month programme? What would help to facilitate capacity? (environmental context and resources) |
| - Given people were trained and happy to do this what barriers might there be to putting it in place from your point for view? Does this differ between a 12 week and 12-month commitment? (environmental context and resources) |
| - Practical/resource |
| - From staff, patients, systems? |
| - What do you think would happen if you did take this on? (for self/patients) |
| - If this did become part of your role is there anything which would make it more likely you’d do it (incentives), what would these look like? (Reinforcement) |
| - Would there be systems that could help monitor if it’s being done, make it easier? (Behavioural regulation) |
| If the exercise programme were put in place what do you think patients would think about it? |
| - Positive/negative reactions |
| - Barriers to attending, |
| - What would make them more likely to attend and maintain their involvement? |
| - Going back to training, if this was to take place, would you be able to undertake training sessions in your current role? |
| - What would be the best way to deliver the training? |
| **Probe* format, length, time of day, location, number of sessions, duration of sessions, practical element with supervision, including videoing?* |
| - Would you be prepared to do things like homework, keep a reflective journal? |
| - If we were to develop a training programme with a view to implementing an intervention would you be interested in taking part? (Intention) |
| - If not, why not? |

**File 1b: Focus group topic guide for healthcare professionals for rehearsal delivery**

**Topics to be include in group discussion following Two-hour basic HCP training (PCa care team training) on exercise and PCa care (work package 2i)**

How do you feel about the training experience?

- Content - Was it understandable? Was it enjoyable?
- Delivery - Was it pitched at the correct Level?
- Was the duration correct? Delivered by right personnel

Did participants feel they had learned something new from the training? What new skills/information do you feel you have learnt from the course?

- probe (different skills targeted)

Do you think there are other topics/skills that would have been useful to include in the training? Were there any aspects of the course which you did not find helpful?

How confident do you feel in being able to use these skills as part of the STAMINA intervention/ in routine practice

- What would increase your confidence?

How motivated do you feel to deliver STAMINA within your team?

- Probe, all members of team
- Anything that could aid motivation

Do you see any barriers to using these skills in practice/ Are there any things which could make it easier?

How do you feel patients will respond to this new approach?

- Helpful?
- Acceptable to patients?

Would you recommend the course to others?

**Reference**

Kirkpatrick, D.L. (1977), Evaluating training programs: Evidence vs proof‟, Training and Development Journal, pp.9-12.

**Additional file 1c: Stakeholder workshop topic guide**

**Topic Guide for two workshops to discuss developing content of the STAMINA interventions for HCPs and the pathways of communication between HCPs and exercise professionals (work package 2iii)** (Adapted from the TIDieR checklist, BMJ 2014 and Murray BMC Medicine 2010)

(Preamble will include presentation of proposed intervention under TIDieR Headings. Although all elements of the intervention will be described and open for comment the primary elements for comment are the HCP elements including HCP behaviours and communication pathways)

Opinions on the proposed content, format and structure of the intervention (HCP, patient and exercise professional elements and communication pathways) (Coherence)

Does the intervention have a clear purpose for all participants (HCPs in the MDT, exercise professional and study patient participants)?

How feasible is the delivery of the intervention?

- Opinions on the mode of delivery of intervention?
- Opinions on the duration of intervention?

Do participants believe the intervention will be put in place (cognitive participation/context)

- What would be peoples’ motivations, barriers, capabilities to put in place?
- Does intervention fit with individuals’ roles? Does the intervention fit with the overall organizational goals of the hospital team and the exercise professionals?

Do participants believe the intervention will bring benefits (and be perceived as advantageous) for patients, for staff and for organisations?

- How will benefits be recognised,
- Ways to facilitate this

How acceptable is the new HCP behaviour likely to be to patients (the ultimate recipients)?

How acceptable is the communication pathway likely to be to patients?

Opinions on the proposed pathways of communication between health care professional and exercise professionals

- What are the best ways of communicating between these two groups?
- What is feasible?
- How acceptable is this communication, to patients?

How might the fidelity of the intervention be promoted?

Will it be clear from the study what effects the intervention has had, will the team be aware of benefits?

Is there learning from related areas that could be helpful here e.g. cardiac rehabilitation.
