# Supplementary material for: The development of a theory and evidence-based intervention to aid implementation of exercise into the prostate cancer care pathway with a focus on healthcare professional behaviour, the STAMINA trial
Source: BMC Health Serv Res. 2021 Mar 25;21:273. doi: 10.1186/s12913-021-06266-x (PMC7992804; doi:10.1186/s12913-021-06266-x)
Supplement: Supplementary file 2 — Additional file 2. Behavioural diagnosis of the seven target behaviours. This file contains an overview of the main barriers to delivery of the seven target behaviours, mapped onto the Theoretical Domains Framework. [file 12913_2021_6266_MOESM2_ESM.docx]

**The development of a theory and evidence-based intervention to aid implementation of exercise into the prostate cancer care pathway with a focus on healthcare professional behaviour, the STAMINA trial**

Rebecca R Turner^1^, Madelynne A Arden^2^_,_ Sophie Reale^1^, Eileen Sutton^3^, Stephanie J C Taylor^4^, Liam Bourke^1^, Diana M Greenfield^5,8^, Dylan Morrissey^6,7^, Janet Brown^8^, Patrick Doherty^9^, Derek J Rosario^1,10^ ,Liz Steed^4^ and on behalf of the STAMINA co-investigators.

^1^ Allied Health Professionals, Radiotherapy and Oncology, Sheffield Hallam University, UK

^2^ Centre for Behavioural Science and Applied Psychology (CeBSAP), Sheffield Hallam University, UK

^3^ Population Health Sciences, University of Bristol, UK

^4^ Institute for Population Health Sciences, Queen Mary, University of London, UK

^5^ Specialised Cancer Services, Sheffield Teaching Hospital NHS Foundation Trust

^6^ Sports and Exercise Medicine, William Harvey Research Institute, School of Medicine and Dentistry, Queen Mary, University of London, London UK

^7^ Physiotherapy Department, Barts Health NHS Trust, London, UK

^8^ Department of Oncology and Metabolism, University of Sheffield, UK

^9^ Department of Health Sciences, University of York, UK

^10^Department of Urology, Sheffield Teaching Hospitals, UK

**Corresponding author:** Liz Steed ([e.a.steed@qmul.ac.uk](mailto:e.a.steed@qmul.ac.uk))

**Additional file 2: Behavioural diagnosis of the seven target behaviours**

| Behaviours | TDF domains | What is needed for change? | Is there a need for change? |
| --- | --- | --- | --- |
| 1. Recognise whether a patient is suitable for exercise | Skills | Not identified in the interviews in relation to this target behaviour | No |
|  | Knowledge | Need the knowledge of whether a patient is suitable for exercise or not | Yes |
|  | Memory, attention, and decision processes | Not identified in the interviews in relation to this target behaviour | No |
|  | Behavioural regulation | Not identified in the interviews in relation to this target behaviour | No |
|  | Social/Professional role and identity | Need to perceive it as part of their role to recognise patients are eligible to exercise | Yes |
|  | Beliefs about capabilities | Need to improve confidence in recognising if patients are eligible for exercise | Yes |
|  | Beliefs about consequences | Not identified in the interviews in relation to this target behaviour | No |
|  | Intentions | Not identified in the interviews | No |
|  | Optimism | Not identified in the interviews | No |
|  | Goals | Not identified in the interviews | No |
|  | Reinforcement | Not identified in the interviews | No |
|  | Emotion | Not identified in the interviews in relation to this target behaviour | No |
|  | Social influences | Not identified in the interviews in relation to this target behaviour | No |
|  | Environmental context and resources | Not identified in the interviews in relation to this target behaviour | No |
| 2. Recommend exercise training at any point within the pathway | Skills | Need behavioural support skills to support patients with exercise. | Yes |
|  | Knowledge | Need the knowledge of the benefits of exercise and awareness of the evidence-based recommendations.  Need the knowledge of the behaviour change skills needed to provide exercise support by all members of the clinical team. | Yes |
|  | Memory, attention, and decision processes | Need to remember to discuss exercise and provide exercise support to patients. | Yes |
|  | Behavioural regulation | Need to develop routines and habits to discuss exercise with patients.  Need for monitoring of this to be in place. | Yes |
|  | Social/Professional role and identity | Need to perceive exercise recommendation to be perceived as part of their role | Yes |
|  | Beliefs about capabilities | Not identified in the interviews in relation to this target behaviour | No |
|  | Beliefs about consequences | Need to believe exercise is beneficial for this patient group.  Need to believe exercise is an important part of patient’s care. | Yes |
|  | Intentions | Not identified in the interviews | No |
|  | Optimism | Not identified in the interviews | No |
|  | Goals | Not identified in the interviews | No |
|  | Reinforcement | Not identified in the interviews | No |
|  | Emotion | Not identified in the interviews in relation to this target behaviour | No |
|  | Social influences | Need to maintain a good relationship with the patient when discussing exercise.  Need to perceive or observe colleagues are providing the same support to patients.  Need to have support from the organisation regarding change that is necessary. | Yes |
|  | Environmental context and resources | More time needed for in-depth discussions around exercise or to develop strategies to discuss exercise under time-pressures. | Yes |
| 3. Discuss barriers and facilitators around exercise training, provide support using BCTs | Skills | Need behavioural support skills to support patients with exercise. | Yes |
|  | Knowledge | Need the knowledge of the behaviour change skills needed to provide exercise support by all members of the clinical team. | Yes |
|  | Memory, attention, and decision processes | Need to remember to discuss exercise and provide exercise support to patients. | Yes |
|  | Behavioural regulation | Not identified in the interviews in relation to this target behaviour | No |
|  | Social/Professional role and identity | Need to perceive discussing barriers and facilitators to exercise and providing behavioural support is part of their tole. | Yes |
|  | Beliefs about capabilities | Not identified in the interviews in relation to this target behaviour | No |
|  | Beliefs about consequences | Need to understand the importance of providing behavioural support. | Yes |
|  | Intentions | Not identified in the interviews | No |
|  | Optimism | Not identified in the interviews | No |
|  | Goals | Not identified in the interviews | No |
|  | Reinforcement | Not identified in the interviews | No |
|  | Emotion | Not identified in the interviews in relation to this target behaviour | No |
|  | Social influences | Need to perceive or observe colleagues providing behavioural support | Yes |
|  | Environmental context and resources | More time needed for in-depth discussions around exercise or to develop strategies to discuss exercise under time-pressures. | Yes |
| 4. Provide patient with information pack and materials | Skills | Not identified in the interviews in relation to this target behaviour | No |
|  | Knowledge | Need the knowledge of the patient materials to hand out to patients. | Yes |
|  | Memory, attention, and decision processes | Need to remember to give the information packs to patients. | Yes |
|  | Behavioural regulation | Not identified in the interviews in relation to this target behaviour | No |
|  | Social/Professional role and identity | HCPs often give out patient materials to patients as part of their role | No |
|  | Beliefs about capabilities | Not identified in the interviews in relation to this target behaviour | No |
|  | Beliefs about consequences | Need to perceive the information for the patients as beneficial. | Yes |
|  | Intentions | Not identified in the interviews | No |
|  | Optimism | Not identified in the interviews | No |
|  | Goals | Not identified in the interviews | No |
|  | Reinforcement | Not identified in the interviews | No |
|  | Emotion | Not identified in the interviews in relation to this target behaviour | No |
|  | Social influences | Not identified in the interviews in relation to this target behaviour | No |
|  | Environmental context and resources | Need to ensure the resources are available to give to patients | Yes |
| 5. Make referral for exercising training | Skills | Need physical skills to make an exercise referral | Yes |
|  | Knowledge | Need to have an awareness of the processes for exercise referral. | Yes |
|  | Memory, attention, and decision processes | Need to remember to make an exercise referral.  Need to believe patients will want to take part in exercise. | Yes |
|  | Behavioural regulation | To monitor number of exercise referrals made by each HCP within a clinical team | Yes |
|  | Social/Professional role and identity | For HCPs to perceive making an exercise referral is part of their role | No |
|  | Beliefs about capabilities | Not identified in the interviews in relation to this target behaviour | No |
|  | Beliefs about consequences | Need for HCPs to trust exercise professionals.  Need to believe patients will want to take part in exercise. | Yes |
|  | Intentions | Not identified in the interviews | No |
|  | Optimism | Not identified in the interviews | No |
|  | Goals | Not identified in the interviews | No |
|  | Reinforcement | Not identified in the interviews | No |
|  | Emotion | Need to have a positive view about referring patients to an exercise referral scheme | Yes |
|  | Social influences | Not identified in the interviews in relation to this target behaviour | No |
|  | Environmental context and resources | Need access to an exercise referral scheme.  Exercise referral needs to be a simple process.  More time needed for exercise referral or to develop strategies to carry out exercise referrals under time-pressures. | Yes |
| 6. Read and interpret exercise progress report | Skills | Need the skills to access the progress report | Yes |
|  | Knowledge | Need to have an awareness of the processes for the progress report. | Yes |
|  | Memory, attention, and decision processes | Need to remember to access the progress report prior to consultation with patient. | Yes |
|  | Behavioural regulation | Not identified in the interviews in relation to this target behaviour | No |
|  | Social/Professional role and identity | Interpreting and reading tests results is already part of HCP roles | No |
|  | Beliefs about capabilities | HCPs already interpret and read test results as part of their role | No |
|  | Beliefs about consequences | HCPs thought feedback on progress would be beneficial | No |
|  | Intentions | Not identified in the interviews | No |
|  | Optimism | Not identified in the interviews | No |
|  | Goals | Not identified in the interviews | No |
|  | Reinforcement | Not identified in the interviews | No |
|  | Emotion | Not identified in the interviews in relation to this target behaviour | No |
|  | Social influences | Not identified in the interviews in relation to this target behaviour | No |
|  | Environmental context and resources | Need to have the time to access the report and read it prior to consultations | Yes |
| 7. Provide feedback to the patient on the exercise progress report | Skills | Need behavioural support skills to support patients with exercise. | Yes |
|  | Knowledge | Need the knowledge of the behaviour change skills needed to provide exercise support by all members of the clinical team. | Yes |
|  | Memory, attention, and decision processes | Need to remember to provide feedback and exercise support to patients. | Yes |
|  | Behavioural regulation | Not identified in the interviews in relation to this target behaviour | No |
|  | Social/Professional role and identity | Need to perceive discussing barriers and facilitators to exercise and providing behavioural support is part of their tole. | Yes |
|  | Beliefs about capabilities | Not identified in the interviews in relation to this target behaviour | No |
|  | Beliefs about consequences | Need to understand the importance of providing behavioural support. | Yes |
|  | Intentions | Not identified in the interviews | No |
|  | Optimism | Not identified in the interviews | No |
|  | Goals | Not identified in the interviews | No |
|  | Reinforcement | Not identified in the interviews | No |
|  | Emotion | Not identified in the interviews in relation to this target behaviour | No |
|  | Social influences | Need to perceive or observe colleagues providing behavioural support | Yes |
|  | Environmental context and resources | More time needed for in-depth discussions around exercise or to develop strategies to discuss exercise under time-pressures. | Yes |
