# Supplementary material for: The development of a theory and evidence-based intervention to aid implementation of exercise into the prostate cancer care pathway with a focus on healthcare professional behaviour, the STAMINA trial
Source: BMC Health Serv Res. 2021 Mar 25;21:273. doi: 10.1186/s12913-021-06266-x (PMC7992804; doi:10.1186/s12913-021-06266-x)
Supplement: Supplementary file 3 — Additional file 3. Feedback on the healthcare professional intervention following the rehearsal delivery. This file provides feedback on the intervention (training package) following rehearsal delivery to healthcare professionals. Feedback is collated into key themes and mapped onto the APEASE criteria. [file 12913_2021_6266_MOESM3_ESM.docx]

**The development of a theory and evidence-based intervention to aid implementation of exercise into the prostate cancer care pathway with a focus on healthcare professional behaviour, the STAMINA trial**

Rebecca R Turner^1^, Madelynne A Arden^2^_,_ Sophie Reale^1^, Eileen Sutton^3^, Stephanie J C Taylor^4^, Liam Bourke^1^, Diana M Greenfield^5,8^, Dylan Morrissey^6,7^, Janet Brown^8^, Patrick Doherty^9^, Derek J Rosario^1,10^ ,Liz Steed^4^ and on behalf of the STAMINA co-investigators.

^1^ Allied Health Professionals, Radiotherapy and Oncology, Sheffield Hallam University, UK

^2^ Centre for Behavioural Science and Applied Psychology (CeBSAP), Sheffield Hallam University, UK

^3^ Population Health Sciences, University of Bristol, UK

^4^ Institute for Population Health Sciences, Queen Mary, University of London, UK

^5^ Specialised Cancer Services, Sheffield Teaching Hospital NHS Foundation Trust

^6^ Sports and Exercise Medicine, William Harvey Research Institute, School of Medicine and Dentistry, Queen Mary, University of London, London UK

^7^ Physiotherapy Department, Barts Health NHS Trust, London, UK

^8^ Department of Oncology and Metabolism, University of Sheffield, UK

^9^ Department of Health Sciences, University of York, UK

^10^Department of Urology, Sheffield Teaching Hospitals, UK

**Corresponding author:** Liz Steed ([e.a.steed@qmul.ac.uk](mailto:e.a.steed@qmul.ac.uk))

**Additional file 3: A summary of feedback on the healthcare professional intervention following the rehearsal delivery**

| **Key theme** | **Feedback** | **Example quote** | **Is a change necessary?**  **Does it meet the APEASE criteria?** | **Impact upon intervention** |
| --- | --- | --- | --- | --- |
| Content of the training package | 1. Including patient case studies in the training was suggested. | *"You want us to tell clinicians, patients, if we can picture what we’re prescribing in talking to them, then we’re much more animated, much more bought into it."* **FG3** | Yes, meets APEASE criteria. | Patient case studies and scenarios will be used throughout the training session. |
|  | 2. To include more task-orientated activities | *"So, we’re task orientated, but if we don’t have an outlet or an outcome that improves it, we tend to ignore it, which is probably a bad thing.”* **FG3** | Yes, meets APEASE criteria. | The inclusion of task-orientated activities will be used throughout the training session. |
|  | 3. A simple message needs to be conveyed to the HCPs about what is expected of them and what is required for the study. | *"Because the classic NHS, which is the environment we are working in all the time, is we want you to do something different. This is what we want you to do. So, we are quite open at having our pathways turned upside down overnight by someone telling us to do something different, so we don’t have a problem with that. We just want to know what we’ve got to do basically."* **FG3** | Yes, meets APEASE criteria. | A clear message of the HCP's roles will be presented in the training session. This will be referred to throughout the training, to ensure HCPs are clear about their new roles. |
|  | 4. There were mixed opinions on including role play within the training session, as some found it 'intimidating', whereas others enjoyed taking part in role play activities as part of training. | *"Yeah, especially if it’s in front of your colleagues (role play), it is quite intimidating. It does not make it very easy."* **FG1**  *"So yeah, I get very much in it. I get that for some people roleplay, they get nervous about being up in front of people. But I personally think it adds a bit of fun to it."* **FG2** | Yes, meets APEASE criteria. | Whilst there was concerns about role play, using role play is a useful tool for skill-based learning. Therefore, the training will include aspects of role play. |
| Duration of training package | The training was posed as being too long and would be difficult to have HCPs to attend. | *"It was good. If I am honest it’s too slow and too long for busy people over here. But you want, as urologists you have got two basically, so if you want to get more people."* **FG3** | Yes, meets APEASE criteria. | A Level one introductory session (60 minutes) will be offered for all the clinical team and a Level two advanced (2.5 hours) will be offered to the all the clinical team but essential for the keyworkers. |
|  | However, it was perceived that if training were reduced, HCPs would not engage. | *"Yeah, 20 minutes long, you won’t have people on board."* **FG1** | Yes, meets APEASE criteria. |  |
| Mode of delivery of training | Face-to-face training was preferred over online training as questions can be asked face-to-face. It was also stated online training tends to get left till the last minute. | *"I’m a bit old school, I like face-to-face things, but that’s because I’ve always got questions. I’ve always got to ask questions."* **FG2**  *"Whenever we get anything online, bearing in mind the number of emails we get every day, it tends to get left. A bit like when we do mandatory training, we leave it until when you have to do it."* **FG1** | Yes, meets APEASE criteria. | The training will ideally be delivered face-to-face for interactive skills-based learning. |
| Barriers to the intervention | Difficulties were identified in being able to release HCPs, particularly staff nurses, was discussed. | *"I think like I said before, from a band 5 perspective I think you’re going to find that really difficult. I do not think we can afford that amount of time out of clinical duties for one trial. From a research nurse point of view that is fine, but from a clinical staff nurse, band 5 point of view I don’t think we can be spared, especially with winter pressures at the moment."*  **FG4** | Yes, meets APEASE criteria. | Difficulties in HCPs attending the training sessions were discussed due to the pressures of the NHS. Therefore, possibly offering the following alternatives may help to resolve some of these issues.   1. Offer at least 8-12 weeks' notice for the clinical team. 2. Ensure senior managers and clinicians are bought into the concept and support the project. |
