# Supplementary material for: The development of a theory and evidence-based intervention to aid implementation of exercise into the prostate cancer care pathway with a focus on healthcare professional behaviour, the STAMINA trial
Source: BMC Health Serv Res. 2021 Mar 25;21:273. doi: 10.1186/s12913-021-06266-x (PMC7992804; doi:10.1186/s12913-021-06266-x)
Supplement: Supplementary file 4 — Additional file 4. Feedback on the healthcare professional intervention from the stakeholder workshop. This file provides feedback on the intervention (training package) following the presentation to stakeholders and roundtable discussions. Feedback is collated into key themes, mapped onto the Normalisation Process Theory and APEASE criteria. [file 12913_2021_6266_MOESM4_ESM.docx]

**The development of a theory and evidence-based intervention to aid implementation of exercise into the prostate cancer care pathway with a focus on healthcare professional behaviour, the STAMINA trial**

Rebecca R Turner^1^, Madelynne A Arden^2^_,_ Sophie Reale^1^, Eileen Sutton^3^, Stephanie J C Taylor^4^, Liam Bourke^1^, Diana M Greenfield^5,8^, Dylan Morrissey^6,7^, Janet Brown^8^, Patrick Doherty^9^, Derek J Rosario^1,10^ ,Liz Steed^4^ and on behalf of the STAMINA co-investigators.

^1^ Allied Health Professionals, Radiotherapy and Oncology, Sheffield Hallam University, UK

^2^ Centre for Behavioural Science and Applied Psychology (CeBSAP), Sheffield Hallam University, UK

^3^ Population Health Sciences, University of Bristol, UK

^4^ Institute for Population Health Sciences, Queen Mary, University of London, UK

^5^ Specialised Cancer Services, Sheffield Teaching Hospital NHS Foundation Trust

^6^ Sports and Exercise Medicine, William Harvey Research Institute, School of Medicine and Dentistry, Queen Mary, University of London, London UK

^7^ Physiotherapy Department, Barts Health NHS Trust, London, UK

^8^ Department of Oncology and Metabolism, University of Sheffield, UK

^9^ Department of Health Sciences, University of York, UK

^10^Department of Urology, Sheffield Teaching Hospitals, UK

**Corresponding author:** Liz Steed ([e.a.steed@qmul.ac.uk](mailto:e.a.steed@qmul.ac.uk))

**Additional file 4: Feedback on the healthcare professional intervention from the stakeholder workshop: mapped onto the Normalisation Process Theory and the APEASE criteria**

| **Key themes** | **Feedback** | **Is a change necessary?**  **Does it meet the APEASE criteria?** | **Impact upon intervention** |  |
| --- | --- | --- | --- | --- |
| *Coherence*: Sense-making work that people do individually or collectively | Training should highlight the evidence-base to support the wider intervention. | Yes, meets the APEASE criteria. | Further information about the evidence behind the importance of exercise for men with prostate cancer on ADT will be added to the training and training materials. |  |
|  |  |  |  |  |
|  |  |  |  |  |
|  |  |  |  |  |
| *Cognitive Participation*: Relational work that people do to build and sustain a community of practice | Training should include more task-based exercises, clear key roles for HCPs and patient case studies. | Yes, meets the APEASE criteria. | These elements already exist within the training package but additional exercises including patient case studies will be added. Additionally, key roles for HCPs will be presented from the start and throughout and clinic prompts highlighting key roles will be developed. |  |
| *Collective Action*: Operational work that people do to enact a set of practices | Training needs to be supported by the organisation but flexible to fit within the context of the NHS. | Yes, meets the APEASE criteria. | Two dates for training will be given for NHS sites, training will take place during working hours and HCPs will be given advance notice of the training session dates, with support from management. |  |
| Reflexive monitoring:  Appraisal work people do to assess and understand the ways a new set of practices affect them and the others around them | Training needs to be delivered face to face in a clinical team setting to aid problem solving and action planning as a team. | Yes, meets the APEASE criteria. | These elements already exist within the training package but additional team action planning and problem solving will be added. Ensuring training is delivered as a clinical team is a crucial element for this training and will be maintained. |  |
