# Supplementary material for: The development of a theory and evidence-based intervention to aid implementation of exercise into the prostate cancer care pathway with a focus on healthcare professional behaviour, the STAMINA trial
Source: BMC Health Serv Res. 2021 Mar 25;21:273. doi: 10.1186/s12913-021-06266-x (PMC7992804; doi:10.1186/s12913-021-06266-x)
Supplement: Supplementary file 5 — Additional file 5. Template for Intervention Description and Replication (TIDieR) of the healthcare professional intervention. This file provides an overview of the healthcare professional intervention (training package). [file 12913_2021_6266_MOESM5_ESM.docx]

**The development of a theory and evidence-based intervention to aid implementation of exercise into the prostate cancer care pathway with a focus on healthcare professional behaviour, the STAMINA trial**

Rebecca R Turner^1^, Madelynne A Arden^2^_,_ Sophie Reale^1^, Eileen Sutton^3^, Stephanie J C Taylor^4^, Liam Bourke^1^, Diana M Greenfield^5,8^, Dylan Morrissey^6,7^, Janet Brown^8^, Patrick Doherty^9^, Derek J Rosario^1,10^ ,Liz Steed^4^ and on behalf of the STAMINA co-investigators.

^1^ Allied Health Professionals, Radiotherapy and Oncology, Sheffield Hallam University, UK

^2^ Centre for Behavioural Science and Applied Psychology (CeBSAP), Sheffield Hallam University, UK

^3^ Population Health Sciences, University of Bristol, UK

^4^ Institute for Population Health Sciences, Queen Mary, University of London, UK

^5^ Specialised Cancer Services, Sheffield Teaching Hospital NHS Foundation Trust

^6^ Sports and Exercise Medicine, William Harvey Research Institute, School of Medicine and Dentistry, Queen Mary, University of London, London UK

^7^ Physiotherapy Department, Barts Health NHS Trust, London, UK

^8^ Department of Oncology and Metabolism, University of Sheffield, UK

^9^ Department of Health Sciences, University of York, UK

^10^Department of Urology, Sheffield Teaching Hospitals, UK

**Corresponding author:** Liz Steed ([e.a.steed@qmul.ac.uk](mailto:e.a.steed@qmul.ac.uk))

### Additional file 5: Template for Intervention Description and Replication (TIDieR) of the healthcare professional intervention

| **Name** | Healthcare professional training package: Integrating exercise recommendations into clinical care |
| --- | --- |
| **Why** | Healthcare professionals (HCPs) do not routinely discuss exercise with men with prostate cancer on ADT and report several barriers as to why. Therefore, a training package is required to support HCPs in recommending exercise, providing behavioural support and exercise in line with recent NICE recommendations NG131 1.4.19. |
| **What** | **Module one: An overview of the project (Level one)**  This module aims to introduce the facilitators to the clinical team, give an overview of the training package and an overview of the project. NICE NG131 1.4.19 recommendations will be introduced here.  **Module two: Prostate cancer and exercise – the evidence base (Level one)**  This module will give an overview of the benefits of exercise for men with prostate cancer on Androgen Deprivation therapy.  **Module three: Discussion exercise as a healthcare professional (Level one)**  This module will identify clinical roles within the team to aid the implementation of the NICE recommendations. How to discuss exercise and lifestyle with this patient group will also be introduced. Common assumptions made by HCPs about patients’ capabilities to exercise will aimed to addressed.  **Module four: Skills to supporting people with exercise (Level two)**  This module will provide HCPs with the appropriate skills in terms of behaviour change techniques to use to support this patient group with exercise. Role-play and group tasks will be included within this module.  **Module five: The exercise referral pathway and communication pathway (Level two)**  This module will provide HCPs with the information of how to make referrals for exercise, what information to hand out to patients and how secure communication will take place with NH and the NHS.  **Module six: Follow-up (Level two)**  This module will provide HCPs with the appropriate skills to provide exercise support at follow-up. Role-play and group tasks will be included within this module.  Level one is designed for all HCPs who see men with prostate cancer on ADT, it includes three modules, see Table 5. It provides an overview of the recommendations, evidence, and new behaviours/roles for the HCPs. It also provides support how to deliver three of these behaviours (recommendation, referral and recognising a patient as suitable). It is thought all HCPs should be able to deliver these behaviours. Level two is designed for the key workers or HCPs involved in follow-up, who are likely to have more time with patients to discuss exercise. This Level includes three modules also and is predominantly behavioural skill training. It provides task-based exercises, including role-play and feedback and focuses on the delivery of exercise support, providing patient with information packs and materials and discussing exercise at follow-up. |
| **Who provided** | Researcher/Health Psychologist |
| **How** | Face-to-face in small groups of up to 8 |
| **Where** | On site at the hospital or at a university building |
| **When and how much?** | Half day training and module 6 is repeated at 8-12 weeks |
| **Tailoring** | Depending on how follow-up care is delivered at the specific NHS site determines which HCPs should attend this training. |
| **Modifications** | Face-to-face, to be delivered on NHS site or locally to NHS site. |
| **How well** | Process measures, acceptability and fidelity will be measured. |
